# Eco-Friendly β-cyclodextrin and Linecaps Polymers for the Removal of Heavy Metals

**DOI:** 10.3390/polym11101658

**Published:** 2019-10-11

**Authors:** Alberto Rubin Pedrazzo, Alessandra Smarra, Fabrizio Caldera, Giorgia Musso, Nilesh Kumar Dhakar, Claudio Cecone, Asma Hamedi, Ilaria Corsi, Francesco Trotta

**Affiliations:** 1 Department of Chemistry, University of Torino, via P. Giuria 7, 10125 Torino, Italy; alberto.rubinpedrazzo@unito.it (A.R.P.); cuscuta@virgilio.it (A.S.); giorgia.musso@gmail.com (G.M.); nileshkumar.dhakar@unito.it (N.K.D.); claudio.cecone@unito.it (C.C.); hamedi.asma66@yahoo.com (A.H.); francesco.trotta@unito.it (F.T.); 2 Department of Physics, Faculty of Science, Yazd University, Yazd 89195741, Iran; 3 Department of Physical, Earth and Environmental Sciences, University of Siena, via Mattioli 4, 53100 Siena, Italy; ilaria.corsi@unisi.it

**Keywords:** heavy metal adsorption, nanosponge, β-cyclodextrin, linecaps, crosslinked polymers, citric acid polymers

## Abstract

Environment-friendly nanosponges, having a high content of carboxyl groups, were synthesized by crosslinking β-cyclodextrin and linecaps, a highly soluble pea starch derivative, with citric acid in water. Additionally, pyromellitic nanosponges were prepared by reacting β-cyclodextrin and linecaps with pyromellitic dianhydride in dimethyl sulfoxide and used in comparison with the citric nanosponges. After ion-exchange of the carboxyl groups H^+^ with sodium ions, the ability of the nanosponges to sequester heavy metal cations was investigated. At a metal concentration of 500 ppm, the pyromellitate nanosponges exhibited a higher retention capacity than the citrate nanosponges. At lower metal concentrations (≤50 ppm) both the citrate and the pyromellitate nanosponges showed high retention capacities (up to 94% of the total amount of metal), while, in the presence of interfering sea water salts, the citrate nanosponges were able to selectively adsorb a significantly higher amount of heavy metals than the pyromellitate nanosponges, almost double in the case of Cu^2+^.

## 1. Introduction

In the treatment of wastewaters, the removal of heavy metals represents a major concern [1,2]. Unlike organic pollutants, heavy metals cannot be destroyed. Once released into the environment, they are easily absorbed by organisms and accumulated in their tissues. As we climb the food chain, the concentration of heavy metals grows rapidly. Being at the top of the food pyramid, humans are highly exposed to the toxic and sometimes even lethal effects of heavy metals. The first symptoms deriving from exposure to high concentration of heavy metals include allergic reactions, mental disability, dementia, depression, insomnia, vision problems and liver and kidney diseases [3,4]. Recently, the World Health Organization (WHO) identified Pb, As, Cu, Cr, Zn and Cd as the primary toxicity-generating elements for live organisms [5]. Under this scenario, the development of environment-friendly and sustainable filtering substrates able to efficiently bind such metals and remove them from wastewaters, before they spread through the ecosystem, is a priority challenge.

Several techniques, based on different physicochemical processes, can be exploited to achieve metal detoxification, including chemical precipitation [6,7], ion-exchange [8,9], membrane filtration (such as ultra- and nanofiltration, electrodialysis and reverse osmosis) [10], floatation [11], coagulation [12], electrochemical methods [13] and biological and chemical adsorption [14,15]. Currently, wastewaters are purified from metals and organic pollutants mainly by treatment with precipitating agents, membranes, ion-exchange resins, activated carbon and biological agents [16]. Given the high price and technological issues that are associated with the large-scale use of these materials, alternative sorbents, having higher efficiency or lower cost, are being investigated. The list of potential substitutes includes silica-based compounds [17], biomaterials [18,19], zeolites [20], clays [21], polymers [22], carbon materials from alternative sources [23,24,25] and several inexpensive waste materials deriving from industrial and agricultural activities [26,27]. 

Recently, Li et al. developed an environment-friendly chitosan persimmon tannin biocomposite to sequester Pb^2+^ ions. The adsorption was dependent on pH, initial concentration and temperature. When tested on a 200 ppm Pb^2+^ solution at pH 4.5, the maximum adsorption capacity, expressed as mg of adsorbed ion per g of adsorbent, was 179.3 mg/g at 323 K and 143.8 mg/g at 303 K [28].

Yang et al. proposed a poly(ethylene glycol) modified with graphene oxide for the adsorption of several metals (including rare earth elements, such as Y^3+^ and Er^3+^) under different operative conditions (i.e., temperature, initial concentrations, etc.). The highest adsorption efficiency was 204.50, 48.04 and 80.48 mg/g in the case of Pb^2+^, Cu^2+^ and Cd^2+^, respectively [29].

Cellulose-based nanocrystals and nanofibers were developed by Corsi et al. and used as adsorbents to capture heavy metals. Tests performed at room temperature and neutral pH on 150 ppm metal solutions revealed adsorption efficiencies of 84, 77, 101 and 160 mg/g in the case of Cu^2+^, Cd^2+^, Zn^2+^ and Pb^2+^ [30].

Hitherto, porous materials produced from renewable and low-cost sources, such as cellulose [31,32], chitin [33] and starch [34] are one of the most promising class of sorbents.

Cyclodextrins (CDs) and amylose are starch derivatives well known for their complexation properties, which arise from their peculiar structural features. The internal cavities of CDs and amylose helices offer suitable accommodation sites for hydrophobic and weakly hydrophilic guest molecules. In this way, inclusion complexes are formed [35,36]. Nevertheless, in order to efficiently bind metal cations, dextrins must be chemically modified by addition of acidic functional groups. By reacting the hydroxyl groups of dextrins with proper bi- or polyfunctional monomers, bearing free acidic groups that might undergo deprotonation in aqueous media, negatively charged insoluble polymers can be obtained. These polymers, often named nanosponges (NSs) in reference to their intrinsic porosity, have the advantage of being able to form complexes with both organic molecules and metal cations [37,38]. Moreover, NSs can be easily separated from treated water and recovered by simple filtration, being insoluble in all solvents or even compacted within filter cartridges.

**Figure 1:**
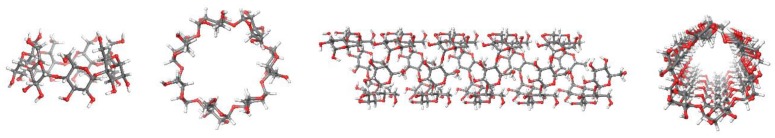
β-cyclodextrin (β-CD) and amylose structure. From left to right, side view and front view of β-CD, side and front view of a 30 glucose units amylose chain.

In this paper, β-CD and a linear starch derivative, i.e., linecaps (LC), were used as building blocks for the preparation of NSs. Within the CDs family, β-CD is the most used one, thanks to its lower cost and medium sized cavity (Figure 1), which can include a broad range of guest molecules. Whereas LC is a pea starch derivative, that is produced and marketed by Roquette Frères as a taste-masking and solubility-enhancement agent, having an average molecular weight of 12 kDa and a content of amylose (Figure 1) of approximately 40% [39].

In previous studies by Berto et al. a dextrin-based NS, prepared from β-CD and pyromellitic dianhydride (PMDA) with a molar ratio PMDA/β-CD = 8, was tested as an innovative metal adsorbent. Although the NS showed a remarkable ability to bind metal ions, such as Al^3+^, Cu^2+^, Zn^2+^, Pd^2+^, Cd^2+^ and U^4+^, the synthesis of the NS involved the use of an organic solvent, i.e., dimethyl sulfoxide, and triethylamine as catalyst [40,41].

The mechanism of adsorption of cations by dextrin polymers bearing carboxyl groups is mainly related to the presence of negative charges in the polymeric structure, that can form complexes with metals. However, cyclodextrin molecules play a synergistic role. As discussed by Prochowicz et al., plain cyclodextrin can interact with transition metals, showing a surprising flexibility in the coordination arrangement. Primary and secondary hydroxyl groups can act as coordination sites to chelate metal ions. Moreover, each CD molecule can coordinate more than one ion and form both homometallic and heterometallic complexes [40,41,42].

In the work presented here, safe and sustainable NSs were prepared by reacting β-CD and LC with citric acid in water, using sodium hypophosphite monohydrate as catalyst [43,44]. The so obtained NSs were used for the adsorption and removal of heavy metals from aqueous solutions, in comparison with NSs prepared by crosslinking β-CD and LC with PMDA.

## 2. Materials and Methods

All the chemicals used in this work were purchased from Sigma-Aldrich (Steinheim, Germany) and used as received, with the exception of β-CD and KLEPTOSE® Linecaps DE17, which were kindly gifted by Roquette Frères (Lestrem, France) and desiccated in oven, at 80 °C up to constant weight, before use. Ultrapure water, 0.2 µm filtered, having a resistivity of 18.2 MΩcm, was produced with a Sartorius Arium® pro ultrapure water system.

### 2.1. Synthesis of Pyromellitic Nanosponges

**Figure 2:**
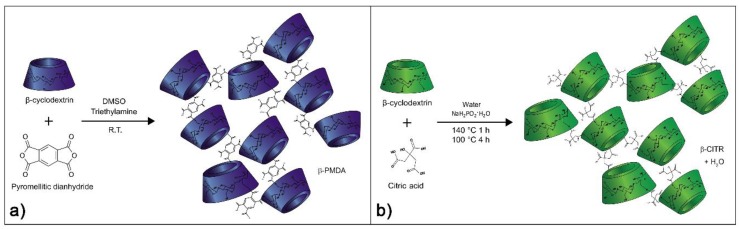
Schematic representation of the synthesis reaction of β-PMDA (**a**) and β-CITR (**b**).

Pyromellitic NSs, named β-PMDA (Figure 2a) and LC-PMDA, were prepared by reacting dextrins with PMDA, through a triethylamine-catalysed polyaddition reaction. Following the previously described procedure [40], 12.26 g of dextrin (β-CD or LC) was dissolved in 50 mL of dimethyl sulfoxide (DMSO) in a 100 mL round bottom flask. Subsequently, 6.3 mL of triethylamine (Et_3_N) and 18.85 g of PMDA were introduced (Table 1). The solution was stirred at room temperature until gelation. 24 h later, the rigid gel was broken with a spatula, ground in a mortar and cleaned with deionized water through Buchner filtration. After a final rinse with acetone, the NS was dried at room temperature.

### 2.2. Synthesis of Citric Acid Nanosponges

Citric acid NSs were synthesized using a polycondensation reaction, between citric acid and dextrin, catalysed by sodium hypophosphite and resulting in the elimination of two molecules of water for each crosslinking bridge (Figure 2b). Specifically, an aqueous solution of monomers was prepared by dissolving 20.00 g of dextrin (β-CD or LC), 3.73 g of sodium hypophosphite monohydrate and 27.09 g of citric acid in 100 mL of deionized water, in a 250 mL beaker. Afterwards, the solution was poured in a 20 cm-diameter crystallizing dish and heated in an oven (Memmert VO500 equipped with a KNF membrane pump) for 1 h at 140 °C and 4 h at 100 °C, under low pressure (~20 mbar). At the end of the reaction, a rigid sponge-like polymer was obtained. The polymer was soaked in deionized water to soften and then it was stirred. After a few minutes, the stirring was stopped and the polymer left to sediment. The supernatant was carefully poured away, in order to remove the soluble and colloidal fractions of the polymer, and replaced with fresh deionized water. This cleaning cycle was repeated five–six times, until a clear and colourless supernatant was observed. Finally, the NS was filtered in a Buchner funnel, rinsed with acetone and left to dry at room temperature. The two NSs prepared with this method are named β-CITR and LC-CITR (Table 2).

### 2.3. Swelling Test

The ability of a NS to swell in water is related to its degree of crosslinking. Generally, the higher the degree of crosslinking, the lower the water uptake. The NSs’ water absorption capacity was studied by performing swelling tests. Specifically, 500 mg of dry NS was introduced in a 7 mL vial placed on a balance and water added dropwise to form a rigid hydrogel. When the amount of water exceeds the maximum volume that the NS is able to absorb, the hydrogel starts flowing as the vial is tilted. The mass of water that allowed a rigid hydrogel to turn into a flowing one was recorded and used to calculate the percentage NS water uptake, according to Equation (1).

(1) $$water\;uptake\;\left(\%\right)=\frac{NS\;hydrogel\;mass\;\left(mg\right)-dry\;NS\;mass\;\left(mg\right)}{dry\;NS\;mass\;\left(mg\right)}*100$$

### 2.4. Preparation of Ion-Exchanged Nanosponges

As confirmed by pH analysis, the addition of citric and pyromellitic NSs to aqueous solutions leads to water acidification. To prevent pH alteration, NSs were subject to ion-exchange with Na^+^ ions. More precisely, 4.00 g of each NS was washed several times in a Buchner funnel with a highly concentrated NaCl solution (i.e. 360 g/L) until the filtrate reached a pH value close to neutrality. Afterwards, the NSs were rinsed with a minimum amount of deionized water, in order to remove the excess of salt, then acetone, and finally they were left to dry at room temperature. The ion-exchanged NSs are named β-PMDA-Na^+^, LC-PMDA-Na^+^, β-CITR-Na^+^ and LC-CITR-Na^+^.

### 2.5. Characterization Study

The synthesized dextrin polymers were characterized by means of Fourier transform infrared analysis in attenuated total reflectance mode (FTIR-ATR) and thermogravimetric analysis (TGA) before and after ion-exchange with Na^+^ ions. Morphology and particle size were studied using scanning electron microscopy (SEM). FTIR-ATR analysis was collected in the 4000–650 cm^−1^ spectral range, using a PerkinElmer Spectrum 100 spectrometer (Shelton, CT, USA). A resolution of 4 cm^−1^ and 8 scan number were set for each analysis. The spectra were then elaborated with PerkinElmer Spectrum software (version 10.03.05) (Shelton, CT, USA). For better comparison, all the spectra were normalized on the main absorption peak of the dextrin units at approximately 1000 cm^−1^.

TGA analysis was performed in a TA Instrument TGA Q500 (New Castle, DE, USA). Approximately 10 mg of sample was weighed in an alumina pan and heated at 10 °C/min from room temperature to 700 °C, under nitrogen flow. The thermograms were elaborated using TA Instruments Universal Analysis 2000 software (version 4.5A) (New Castle, DE, USA).

SEM analysis was performed using an Oxford Instruments Leica Stereoscan 410 microscope (Nussloch, Germany). The NSs were placed on a sample holder covered with a double-sided conductive adhesive tape and observed at a working distance of 10 mm and EHT potential of 8 KV.

### 2.6. Adsorption of Cu^2+^ ions (500 ppm) at Different Contact times

A 500 ppm Cu^2+^ solution was prepared by dissolving the proper amount of CuSO_4_ in ultrapure water (Table 3). Metal adsorption tests were performed by stirring 30 mg of ion-exchanged citric acid NS in 10 mL of metal solution at room temperature. At the initial time and after 30 min, 1 h, 2 h, 3 h, 6 h, 24 h and 30 h the dispersions were centrifuged (for 10 min at 4000 rpm) and the supernatant was analysed using UV–Vis spectroscopy (at 830 nm) with a Perking Elmer UV/Vis Spectrometer Lambda 25 (Shelton, CT, USA). The residual amount of uncomplexed metal was quantified using an external calibration curve in the 20–500 ppm range.

### 2.7. Adsorption of Metal Ions at High Concentration (500 ppm)

Cu^2+^, Zn^2+^, Pb^2+^, Cd^2+^ and Fe^3+^ 500 ppm solutions were prepared by dissolving the proper amount of CuSO_4_, Zn(CH_3_COO)_2_·2H_2_O, PbBr_2_, Cd(CH_3_COO)_2_·2H_2_O and FeCl_3_·6H_2_O in ultrapure water (Table 3). Metal adsorption tests were performed by stirring 10 mg of each ion-exchanged NS in 5 mL of metal solution. After 24 h, the dispersions were centrifuged (4000 rpm, 10 min) and the supernatant was filtered using 0.2 µm polytetrafluoroethylene (PTFE) syringe filters. Then, 1 mL of each solution was added to 1 mL of 65% nitric acid, to prevent precipitation and diluted to 100 mL. Finally, the concentration of uncomplexed Cu^2+^, Zn^2+^, Pb^2+^, Cd^2+^ and Fe^3+^ was quantified by means of inductively coupled plasma mass spectroscopy (ICP-MS) using an AGILENT 7500 CE instrument (Santa Clara, CA, USA) and external calibration method (calibration points: 1, 5, 10, 25, 50 μg/L; each sample solution was suitably diluted to fit within the calibration curve).

### 2.8. Adsorption of Metal Ions at Low Concentration (≤50 ppm)

Cu^2+^ and Zn^2+^ were selected as model metal ions to evaluate the ability of the NSs to adsorb low concentrations of heavy metal pollutants, specifically 50, 5, 1, 0.1 mg/L for Cu^2+^ and 50, 10, 3, 0.5 mg/L for Zn^2+^. 12 mg of each ion-exchanged NS was stirred for 24 h in 15 mL of metal solution, prepared by diluting the 500 ppm Cu^2+^ and Zn^2+^ solutions. Afterwards, the NS was sedimented using centrifugation (4000 rpm, 10 min) and the supernatant was filtered, using 0.2 µm PTFE syringe filters. The residual amount of heavy metal was quantified using ICP-MS analysis, as described above.

### 2.9. Metal Cations Adsorption Tests in Artificial Sea Water

The capacity of the ion-exchanged NSs to remove heavy metals from complex environmental matrices was assessed by performing adsorption tests in the presence of interfering salts. A mimicking solution, having properties and composition similar to sea water, was prepared by mixing several salts, according to the recipe presented in Table 4.

Cu^2+^ and Zn^2+^ 50 ppm solutions were prepared by dissolving the proper amount of CuSO_4_ and Zn(CH_3_COO)_2_·2H_2_O in artificial sea water (Table 3). Cu^2+^ solutions with a concentration of 5, 1, 0.1 ppm and Zn^2+^ solutions with a concentration of 10, 3, 0.5 ppm were prepared from the 50 ppm solutions by dilution with artificial sea water. Subsequently, Cu^2+^ and Zn^2+^ adsorption tests were performed by stirring 12 mg of NS in 15 mL of metal solution. After 24 h, the dispersions were centrifuged (4000 rpm, 10 min) and the supernatant was filtered using 0.2 µm syringe filters. The amount of uncomplexed metal ions was estimated using ICP-MS analysis, as described above.

## 3. Results

Four negatively-charged NSs were synthesized by reacting β-cyclodextrin and linecaps with citric acid and pyromellitic dianhydride. The mass balance, expressed as percentage weight ratio of product to monomers, of β-PMDA, LC-PMDA, β-CITR and LC-CITR was 94%, 92%, 26% and 54%, respectively. A high amount of crosslinker (theoretical molar ratio crosslinker/β-CD = 8) was introduced in order to maximize the content of free carboxyl groups, thus enhancing the complexation ability of the NSs. Additionally, high degrees of crosslinking normally lead to low swellable polymers, which are more suited to water treatment applications than highly swellable polymers. Low swellable polymers can be packed into filter devices, like cartridges, because they undergo only moderate volume variations, upon wetting and drying cycles. In the laboratory work-up, low swellable polymers suit better as well, as they can be easily and rapidly separated from the treated solutions by precipitation and filtration. As expected, the synthesized NSs exhibited a low water uptake (Table 5). Unlike analogous polymers having lower degree of crosslinking (i.e., crosslinker/β-CD molar ratios < 4), which were described to absorb a significant amount of water, up to 2000 wt % [45], the synthesized NSs showed a maximum water uptake below 400 wt %. The higher volume of water absorbed by the citric acid NSs, compared to the PMDA NSs, can be explained by the more hydrophilic nature of the citric acid crosslinking bridges.

The adsorption of metal cations causes the release of H^+^ ions from the NSs free carboxyl groups, which results in alterations of the aqueous solution pH value. To avoid water acidification, all four NSs were ion-exchanged with sodium ions, prior to their use.

### 3.1. Characterization Study

**Figure 3:**
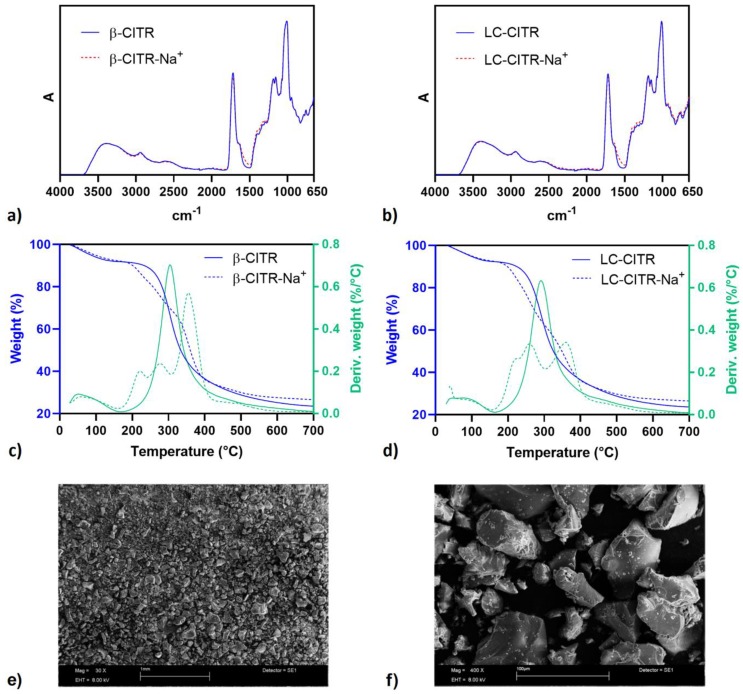
Fourier transform infrared analysis in attenuated total reflectance mode (FTIR-ATR) analysis of β-CITR (**a**) and LC-CITR (**b**) polymers before (solid lines) and after ion-exchange (dashed lines). Thermogravimetric analysis (TGA) of β-CITR (**c**) and LC-CITR (**d**) polymers before (solid lines) and after ion-exchange (dashed lines). The green lines indicate the TGA curve first derivative. Scanning electron microscopy (SEM) images of LC-CITR at 30× (**e**) and 400× (**f**) magnification.

The synthesized dextrin polymers were characterized by means of infrared and thermogravimetric analysis in their acidic form and after ion-exchange with NaCl. The infrared spectra of the citric acid NSs are presented in Figure 3a,b. A broad absorption band, due to the stretching of O–H bonds, is observed in the 3600–3000 cm^−1^ range, close to the C–H bond stretching vibration at approximately 2900 cm^−1^. The strong absorption peak that appears at 1720 cm^−1^ derives from the stretching vibration of C=O bonds belonging to the carboxyl groups of citric acid molecules and the ester bonds between citric acid and dextrin units. While the peaks in the 1200–1000 cm^−1^ region are mostly related to the stretching vibrations of C–O bonds of ether and alcohol groups of dextrin and citric acid units. The infrared spectra of LC and β-CD citric acid polymers after ion-exchange with Na^+^ show a slight increase of the absorption intensity in the 1500–1600 cm^−1^ range, where the stretching vibration of C=O bonds in carboxylate moieties occurs, thus confirming the presence of a higher content of carboxylate groups. Aside from this difference, the infrared spectra of β-CITR and LC-CITR before and after ion-exchange are superimposable. Similarly, PMDA NSs exhibit absorption bands in the 3600–3000 cm^−1^ range, near 2900 cm^−1^, at 1720 cm^−1^ and in the 1250–1000 cm^−1^ region, which are due to O–H, C–H, C=O and C–O stretching vibrations, respectively. After ion-exchange, PMDA-NSs show an intense peak at 1580 cm^−1^ which is associated with the C=O stretching vibration of the carboxylate groups (data not shown).

TGA curves of the citric acid NSs show a two-step weight loss. The first weight loss, of approximately 10%, occurs before 150 °C and is due to the evaporation of adsorbed moisture. Whereas, the thermal degradation of the polymer structure starts in the 150–200 °C range and finishes around 700 °C (Figure 3c,d, blue curves). Compared to pristine β-CD and LC, which start degrading near 300 and 200 °C, respectively, the onset of the NSs degradation occurs at a lower temperature because of the lower thermal stability of the crosslinking unit ester bonds [23]. β-CITR and LC-CITR exhibit a single-peak first derivative of the TGA curve with the maximum rate of weight change near 300 °C, while β-CITR-Na^+^ and LC-CITR-Na^+^ show a more complex weight loss first derivative curve, having three peaks at approximately 220, 270 and 360 °C (Figure 3c,d, green lines). This finding reflects the different chemical composition of the ion-exchanged NSs. As expected, the final residue at 700 °C of the ion-exchanged citrate NSs is higher than the final residue of the pristine citric acid NSs, due to the presence of Na^+^ ions. Consistent results were obtained with the TGA analysis of the pyromellitic NSs (data not shown).

Morphology and particle size of the NSs were evaluated using SEM analysis. All the NSs exhibited irregular morphology and broad size distribution in the micrometre range (Figure 3e,f). Such particle size allows fast precipitation and easy separation of the NSs from the treated solution.

### 3.2. Adsorption of Cu^2+^ Ions (500 ppm) at Different Contact Times

**Figure 4:**
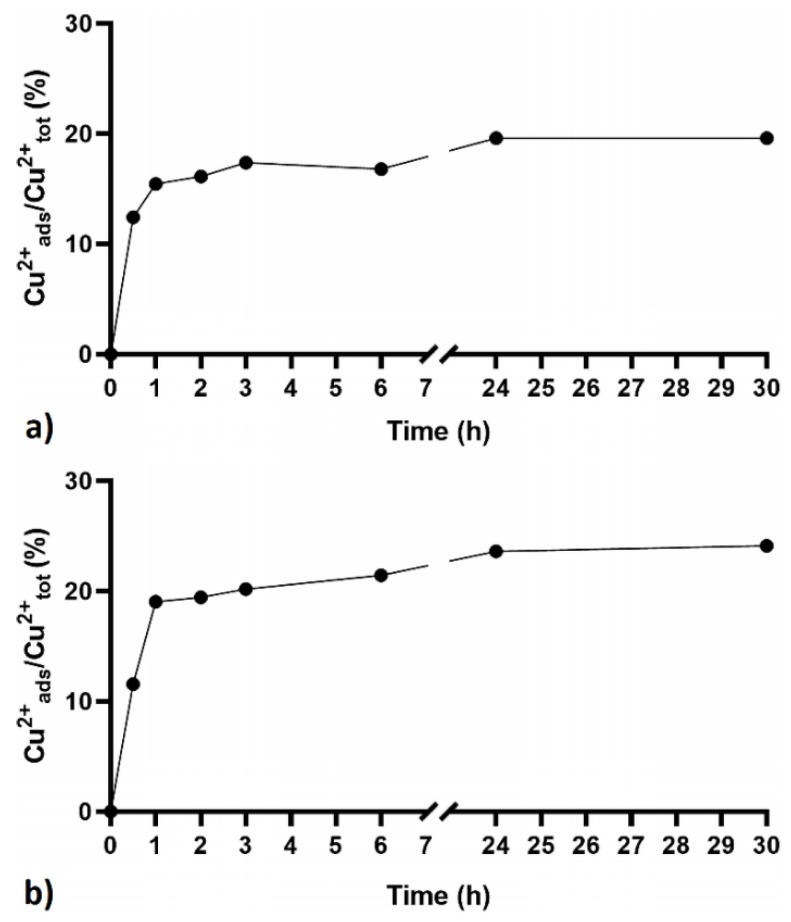
Cu^2+^ adsorption over time by the β-CITR-Na^+^ (**a**) and LC-CITR-Na^+^ (**b**) polymers, added to 500 ppm Cu^2+^ solution.

Preliminary studies of adsorption kinetics were performed in 500 ppm Cu^2+^ solutions. The adsorption of metal ions by the β-CITR-Na^+^ and LC-CITR-Na^+^ NSs was monitored at different times (30 min, 1 h, 2 h, 3 h, 6 h, 24 h and 30 h). As shown in Figure 4, a contact time of 30 min was enough to achieve an adsorption efficiency of 10%. However, the highest capture efficiency and sorption equilibrium were reached after 24 h for both samples. In light of these findings, a contact time of 24 h was selected for the following tests.

### 3.3. Adsorption of Metal Ions at High Concentration (500 ppm)

**Figure 5:**
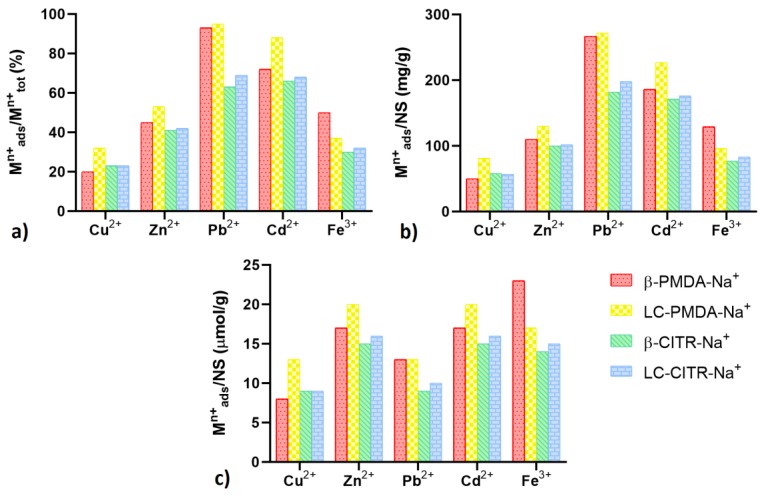
Metal adsorption tests performed in 500 ppm metal solutions. The NSs’ adsorption capacity is expressed as a percentage of the initial amount of metal (**a**), weight ratio adsorbed metal to NS (**b**) and moles of adsorbed metal per gram of NS (**c**).

The capacity of the ion-exchanged NSs of adsorbing heavy metals from highly concentrated solutions (500 ppm) was evaluated. Figure 5a shows the amount of complexed metal ions as a percentage of the total amount. The NSs adsorbed a quantity of metal ions comprised between 20% and 95%. The highest adsorption was observed in the case of Pb^2+^ and Cd^2+^, in particular Pb^2+^ was almost completely removed by the PMDA NSs. The NS LC-PMDA-Na^+^ performed better than the other NSs, except in the case of Fe^3+^, which was more efficiently adsorbed by β-PMDA-Na^+^.

The weight ratio of adsorbed cations to NS is presented in Figure 5b. In accordance with the profile of Figure 5a, the highest performance was observed in the case of Pb^2+^, which was adsorbed by LC-PMDA-Na^+^ up to 272 mg/g. While the lowest uptake was found for Cu^2+^, varying in the range 50–81 mg/g.

The relative adsorption efficiencies change significantly when expressed as moles of complexed metal per gram of NS (Figure 5c). The amount of adsorbed Pb^2+^, owing to its high molar mass, translates into a quantity of moles smaller than the adsorbed moles of Fe^3+^. In terms of moles per gram of NS, the highest metal uptake of 20, 20 and 23 µmol/g were measured treating Zn^2+^, Cd^2+^ and Fe^3+^ solutions, respectively, with PMDA NSs.

Despite having the same crosslinker/dextrin molar ratio, the PMDA NSs possess two carboxyl groups for each PMDA crosslinking unit, while each citric acid crosslinking bridge bears only one free carboxyl group. This may explain the higher metal uptake capacity of the PMDA NSs at 500 ppm metal concentration.

In consideration of the experimental results listed above, no clear correlation can be found between the properties of the metal cations, such as electric charge, diameter, mass, etc., and the adsorption efficiency exhibited by the NSs.

### 3.4. Adsorption of Metal ions at Low Concentration (≤50 ppm)

**Figure 6:**
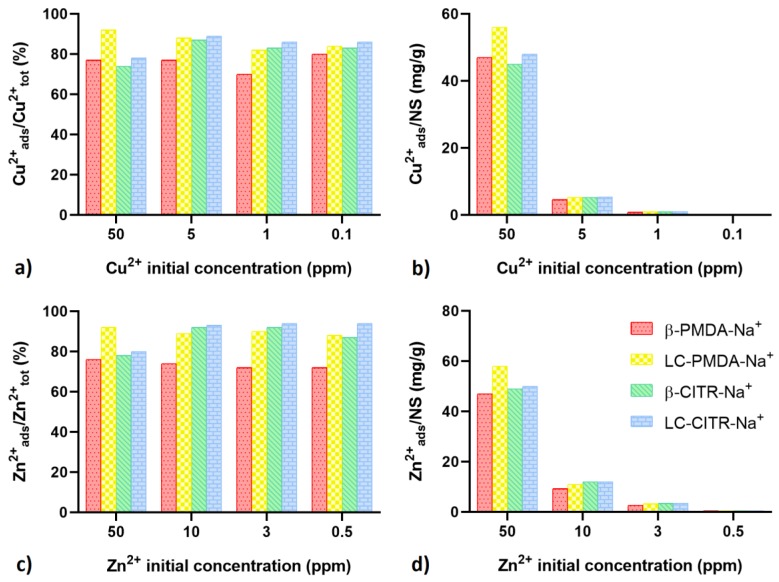
Cu^2+^ (**a**,**b**) and Zn^2+^ (**c**,**d**) adsorption tests on low concentration metal solutions. The amount of complexed metal ions is expressed as a percentage of the initial amount of metal (**a**,**c**) and as moles of adsorbed metal per gram of NS (**b**,**d**).

Adsorption tests were performed in diluted Cu^2+^ and Zn^2+^ solutions, in order to investigate the ability of the NSs to sequester metal cations from low concentrated metal solutions (Figure 6). At concentrations of 50, 5, 1, 0.1 ppm, Cu^2+^ adsorption efficiency was observed to vary in the range from 70% to 92%. Comparing the four NSs, Cu^2+^ at 50 ppm was more efficiently adsorbed by LC-PMDA-Na^+^. Nevertheless, at lower concentrations (i.e., 5, 1 and 0.1 ppm) the citrate NSs showed a similar or even higher Cu^2+^ uptake than LC-PMDA-Na^+^, reaching almost 90% (Figure 6a). In terms of weight ratio of adsorbed metal to NS, an adsorption of 56 mg/g was observed when the 50 ppm Cu^2+^ solution was treated with LC-PMDA-Na^+^, then it dropped below 5.5 mg/g at lower Cu^2+^ concentrations (Figure 6b). Similar results were obtained in the case of Zn^2+^ (Figure 6c,d), which was adsorbed in a range of 72–94%. The citrate NSs performed better than LC-PMDA-Na^+^ at metal concentrations below 50 ppm. Among the synthesized NSs, β-PMDA-Na^+^ exhibited the lowest efficiency, both in the case of Cu^2+^ and Zn^2+^.

### 3.5. Metal Cations Adsorption Tests in Artificial Sea Water

**Figure 7:**
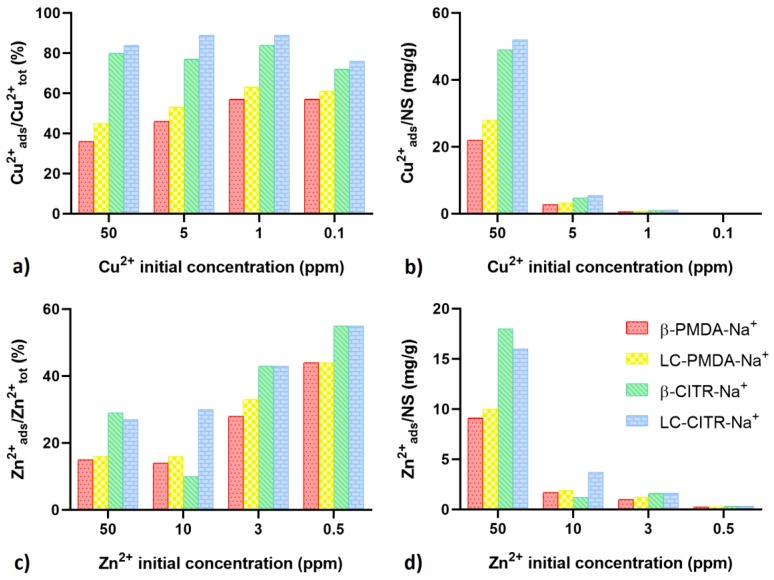
Cu^2+^ (**a**,**b**) and Zn^2+^ (**c**,**d**) adsorption tests on low concentration metal solutions prepared in artificial sea water. The amount of complexed metal ions is expressed as a percentage of the initial amount of metal (**a**,**c**) and as moles of adsorbed metal per gram of NS (**b**,**d**).

The ability of the NSs to selectively adsorb heavy metals from complex matrices was investigated by performing Cu^2+^ and Zn^2+^ adsorption tests in artificial sea water. Contrary to what was observed in ultrapure water, the citrate NSs were significantly more effective than the pyromellitate NSs. When added to 50 ppm Cu^2+^ solutions, the citrate NSs were able to remove around 80–84% of Cu^2+^ ions, equivalent to 49–52 mg of Cu^2+^ per gram of NS. Whereas, the Cu^2+^ uptake of the PMDA NSs plummeted to 36–45% in artificial sea water, corresponding to 22–28 mg per gram of NS. At a lower Cu^2+^ concentration the adsorption capacity of the PMDA NSs increased, albeit remaining below the citrate NSs (Figure 7a,b). The presence of interfering salts had a greater impact on the adsorption of Zn^2+^ for both the NSs, which were observed to complex less than 60% of Zn^2+^ ions, even at the lowest Zn^2+^ concentration (Figure 7c), while in terms of weight ratio, the highest uptake was only 18 mg/g, at a Zn^2+^ concentration of 50 ppm (Figure 7d). However, also in the case of Zn^2+^, the adsorption efficiency of the citrate NSs was higher than the pyromellitate NSs for all test concentrations.

## 4. Conclusions

Nature-friendly NSs were synthesized by crosslinking β-CD and LC with citric acid in water, using sodium hypophosphite monohydrate as catalyst. The NSs were successfully used as heavy metals adsorbents for water detoxification, in comparison with a previously described pyromellitic NS, which was prepared by reacting β-CD and PMDA in DMSO with the addition of triethylamine, and an analogous NS obtained from LC and PMDA. The swelling index of the four NSs confirmed their high degree of crosslinking, hence their high content of carboxyl groups. The tendency of the NSs to acidify wastewaters was decreased by exchanging the carboxyl group H^+^ with Na^+^, prior to use.

Cu^2+^ adsorption kinetics studies indicated that the maximum capture efficiency is reached after 24 h. Nevertheless, 30 minutes contact time was enough to obtain an adsorption efficiency higher than 10%.

Metal adsorption tests were then conducted at different metal concentrations. At 500 ppm, the PMDA NSs exhibited a higher metal retention capacity than the citrate NSs, because of their higher content of carboxyl groups, although no clear correlation was observed between the properties of the metal cations, such as electric charge, diameter, mass, etc., and the adsorption efficiency exhibited by the NSs. At lower metal concentrations (≤50 ppm, tests performed on Cu^2+^ and Zn^2+^ ions), both the citrate and pyromellitate NSs were able to retain a high amount of metal (up to 94%). Cu^2+^ and Zn^2+^ adsorption tests were also conducted in artificial sea water to explore the ability of the NSs to selectively bind heavy metals. The presence of the interfering sea water salts had a greater impact on the adsorption performance of the pyromellitate NSs than the citrate NSs. In particular, the Cu^2+^ uptake by the citrate NSs was almost unchanged.

In light of these findings, the citrate NSs are a promising and sustainable adsorbent material for the removal of heavy metal cations from polluted waters.

## Figures and Tables

**Table 1:** Quantities of chemicals used for the synthesis of pyromellitic dianhydride (PMDA) nanosponges (NSs).

Sample	DMSO	β-CD		LC		Et_3_N		PMDA	
	(mL)	(g)	(mmol)	(g)	(mmol)	(mL)	(mmol)	(g)	(mmol)
β-PMDA	50	12.26	10.8	-	-	6.3	45.4	18.85	86.4
LC-PMDA	50	-	-	12.26	-	6.3	45.4	18.85	86.4

**Table 2:** Quantities of chemicals used for the synthesis of citric acid-based NSs.

Sample	Water	β-CD		LC		NaH_2_PO_2_·H_2_O		Citric Acid	
	(mL)	(g)	(mmol)	(g)	(mmol)	(g)	(mmol)	(g)	(mmol)
β-CITR	100	20.00	17.6	-	-	3.73	35.2	27.08	141.0
LC-CITR	100	-	-	20.00	-	3.73	35.2	27.08	141.0

**Table 3:** Quantities of salt used for the preparation of metal solutions.

Ion	Salt	Weight of Salt for a 100 mL Metal Solution (mg)	
		for 500 ppm Metal Solution in Ultrapure Water	for 50 ppm Metal Solution in Artificial Sea Water
Cu^2+^	CuSO_4_	126	13
Zn^2+^	Zn(CH_3_COO)_2_·2H_2_O	168	17
Pb^2+^	PbBr_2_	89	9
Cd^2+^	Cd(CH_3_COO)_2_·2H_2_O	119	12
Fe^3+^	FeCl_3_·6H_2_O	242	24

**Table 4:** Composition of artificial sea water.

Salt	Concentration		Quantity Introduced in 2 L of Solution (g)
	(mM)	(g/L)	
NaF	0.0452	0.0019	0.004
SrCl_2_·6H_2_O	0.213	0.0567	0.113
H_3_BO_3_	0.323	0.0200	0.040
KBr	0.563	0.0670	0.134
KCl	6.251	0.4660	0.932
CaCl_2_	4.986	0.5533	1.110
Na_2_SO_4_	18.73	2.660	5.320
MgCl_2_	17.97	1.711	3.420
NaHCO_3_	1.58	0.133	0.266
NaCl	473.1	27.650	55.300

**Table 5:** Swelling test results.

Sample	Water Uptake (wt %)
β-PMDA	254
LC-PMDA	116
β-CITR	356
LC-CITR	370
